# Candidates in Astroviruses, Seadornaviruses, Cytorhabdoviruses and Coronaviruses for +1 frame overlapping genes accessed by leaky scanning

**DOI:** 10.1186/1743-422X-7-17

**Published:** 2010-01-25

**Authors:** Andrew E Firth, John F Atkins

**Affiliations:** 1 BioSciences Institute, University College Cork, Cork, Ireland; 2 Department of Human Genetics, University of Utah, Salt Lake City, UT 84112-5330, USA

## Abstract

**Background:**

Overlapping genes are common in RNA viruses where they serve as a mechanism to optimize the coding potential of compact genomes. However, annotation of overlapping genes can be difficult using conventional gene-finding software. Recently we have been using a number of complementary approaches to systematically identify previously undetected overlapping genes in RNA virus genomes. In this article we gather together a number of promising candidate new overlapping genes that may be of interest to the community.

**Results:**

Overlapping gene predictions are presented for the astroviruses, seadornaviruses, cytorhabdoviruses and coronaviruses (families *Astroviridae*, *Reoviridae*, *Rhabdoviridae *and *Coronaviridae*, respectively).

## Background

Overlapping genes (whereby the same nucleotide sequence codes for two or more proteins in different reading frames) are particularly common in RNA viruses, where they may serve as mechanisms to optimize the coding potential of compact genomes, regulate gene expression, or circumvent the host cell's canonical - though not ubiquitous - rule of 'one functional protein per mRNA'. However, such genes can be difficult to detect using conventional gene-finding software.

MLOGD is a gene-finding program which was designed specifically for identifying overlapping coding sequences (CDSs) through the incorporation of explicit models for sequence evolution in multiply-coding regions [1-3]. One caveat is that *de novo *overlapping CDSs are often considerably less conserved than the ancestral genes that they overlap (the ancestral gene is usually the known gene as it tends to be the longer of the two, while the *de novo *gene is often very short). The explicit 'coding signal' of such a CDS may be swamped by the 'coding signal' of the ancestral CDS. Thus there are a number of known overlapping CDSs which MLOGD fails to detect. Another caveat of MLOGD is that, if an overlapping CDS is very short and highly conserved (e.g. due to coding in two different reading frames and perhaps also harbouring an RNA secondary structure for stimulating ribosomal frameshifting into the overlapping CDS; [4]), then there may be too few base variations to obtain a useful signal (either coding or non-coding). On the other hand, for overlapping CDSs that are subject to a reasonable degree of purifying selection and that are not too short, MLOGD can provide a robust detection with just two input sequences provided that they are sufficiently divergent.

A sometimes more sensitive, but generally less specific, approach involves analysis of conservation at synonymous sites within known CDSs. This method is particularly useful when a large and diverse input sequence alignment is available [5,6]. Enhanced conservation may be associated with overlapping functional elements. However such elements may be either coding or non-coding, so additional evidence (e.g. conservation of an overlapping open reading frame and a potential translation mechanism over a sufficiently divergent sequence alignment) is required in order to use this method to identify overlapping CDSs. Care also needs to be taken to discriminate dual-coding sequences from regions of enhanced conservation that may arise from recombination.

Over the past few years we have engaged in a systematic survey of viral genomes for previously undetected overlapping genes. While many of these merit detailed analysis and experimental follow-up (either because they are in important and well-studied viruses or because they involve novel non-canonical translation mechanisms), there remain a miscellany of promising candidate new overlapping genes that we are not currently in a position to follow up experimentally but, nonetheless, may be of interest to the community. The purpose of this article is to communicate five of these candidates.

## Results and Discussion

Candidate new overlapping CDSs were identified using either MLOGD or analysis of conservation at synonymous sites within known CDSs. Some candidates were detected by both methods, while others were only detected by one (candidates only detected by MLOGD are typically those for which only a small number of relevant sequences were available; candidates only detected by synonymous site conservation are typically those for which the overlapping CDS is subject to fairly weak constraints at the amino acid level). In all cases, candidates were closely inspected for a potential translation mechanism in the context of current knowledge of the gene expression strategy of the virus in question. Candidates are summarized in Table 1 and discussed individually in the following subsections. Coding potential graphs and nucleotide sequence alignments showing the initiation contexts for each candidate are presented in the figures. It is interesting to note that all five candidates are in the +1 reading frame relative to the annotated, and most likely ancestral, CDS. In fact the preference for the evolution of *de novo *overlapping genes in the +1 frame, as opposed to the +2 frame, has been previously noted and appears to be related to codon (or dicodon) usage in the ancestral CDS [7]. *Note that, in the following, the name 'ORFX' will be used repeatedly to refer to each candidate overlapping CDS*.

**Table 1:** Summary of candidate overlapping CDSs

genus (RNA)	phylogenetic distribution (GenBank RefSeqs)	length (codons)		**ln(LR)**^**2**^	
*Mamastrovirus* (sgRNA)	Human, porcine and feline astroviruses etc [GenBank: NC_001943]	91-122	>500	NA	5.9 × 10^-28^
*Seadornavirus* (segment 7)	Banna virus, Kadipiro virus etc [GenBank: NC_004204, GenBank: NC_004209]	52-65	31	9.6	2.3 × 10^-10^
*Cytorhabdovirus* (P mRNA)	Lettuce necrotic yellows virus etc [GenBank: NC_007642, GenBank: NC_011532]	97-102	149	22.7	NA
*Gammacoronavirus* (NS6 sgRNA)	Group 3c coronaviruses [GenBank: NC_011548, GenBank: NC_011549, GenBank: NC_011550]	69-89	75*	20.6*	NA
*Alphacoronavirus* (ORF3 sgRNA)	Bat coronaviruses 1A, 1B, HKU8 [GenBank: NC_010436, GenBank: NC_010437, GenBank: NC_010438]	91-95	82*	12.4*	NA

1. Approximate total number of phylogenetically independent base variations in the ORFX region (not including sequences with only partial coverage of ORFX).

2. Total MLOGD 'log likelihood' score (not strictly log likelihood due to non-independence issues - see Ref. [1] for details). Positive values indicate that ORFX is likely to be coding. Candidates that were not detected with MLOGD are indicated with 'NA'. For candidates marked with an '*', the poorly constrained C-terminal region (see Figures 7, 9) was excluded for calculation of *N*_var _and ln(LR).

3. Probability that the degree of conservation at synonymous sites of the known CDS within the whole ORFX region could be obtained under a null model of neutral evolution at synonymous sites. Note that a significant *p*-value here does not necessarily indicate an overlapping CDS, merely an overlapping functional element. Candidates for which the available sequence data are too limited to effectively use this approach are indicated with 'NA'.

### Mamastrovirus (human, porcine, feline astrovirus clade)

Astroviruses have monopartite positive-sense ssRNA genomes and are associated with gastroenteritis and viral diarrhoea in humans and other vertebrates. The non-structural polyprotein (ORF1a and, via ribosomal frameshifting, an ORF1a-ORF1b fusion) are translated from the genomic RNA (gRNA) while the structural polyprotein (ORF2) is translated from a sub-genomic RNA (sgRNA) [8,9]. ORFX overlaps the 5' end of ORF2 in the +1 reading frame. A 112-codon AUG-initiated +1 frame ORF is present in nearly all human astrovirus sequences in GenBank with complete or partial coverage of the ORFX region (~780 sequences; ~150 with full coverage of ORFX; 25 Oct 2009). Just a very small number of sequences are ORFX-defective: one partial sequence contains a premature termination codon, one sequence contains a 16-codon 3' extension, and three sequences have a CUG codon (which may, nonetheless, allow a low level of initiation [10]) instead of an AUG codon at the proposed ORFX initiation site.

**Figure 1:**
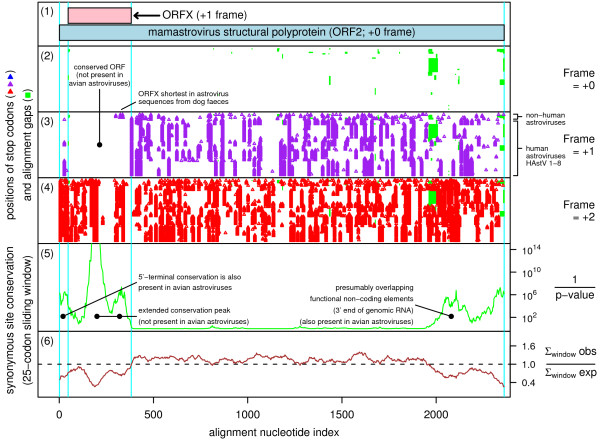
**Coding potential statistics for mamastrovirus (human-porcine-feline astrovirus clade) ORF2 and the overlapping ORFX**. **(1) **Map of the ORF2 region of human astrovirus [GenBank: Z25771], showing the proposed new coding sequence, ORFX, overlapping ORF2 in the +1 reading frame. **(2-6) **Coding potential statistics based on an alignment of 88 mamastrovirus sequences with complete coverage ORF2 (see Methods for accession numbers). For clarity, regions with alignment gaps in the arbitrary reference sequence (viz. Z25771) have been removed (e.g. regions where a single sequence in the alignment has an insertion, resulting in alignment gaps in all the other sequences). **(2-4) **Positions of stop codons in each of the three forward reading frames. The +0 frame corresponds to ORF2 and is therefore devoid of stop codons. Note the conserved absence of stop codons in the +1 frame within the ORFX region. **(5-6) **Conservation at synonymous sites within ORF2 (see [5] for details). (5) depicts the probability that the degree of conservation within a given window could be obtained under a null model of neutral evolution at synonymous sites, while (6) depicts the absolute amount of conservation as represented by the ratio of the observed number of substitutions within a given window to the number expected under the null model. Note the unusually high conservation within the ORFX region.

An AUG-initiated ORF of similar length (91-122 codons) is present in those feline, porcine, dolphin, sea lion and dog astroviruses that cluster with the HAstV human astrovirus clade (see Refs. [11-14] for recent phylogenetic trees). The ORF coincides with a region of enhanced conservation at ORF2-frame synonymous sites (Figure 1). This region of enhanced conservation is not present in the avian astroviruses, which also lack a conserved +1 frame ORF in this region. There is also little evidence for ORFX in other mammalian astrovirus clades (ovine, mink, bat and certain human astroviruses that cluster outside of the classic human-porcine-feline astrovirus clade) - an equivalent of ORFX may be present in some but certainly not all of these astroviruses.

**Figure 2:**
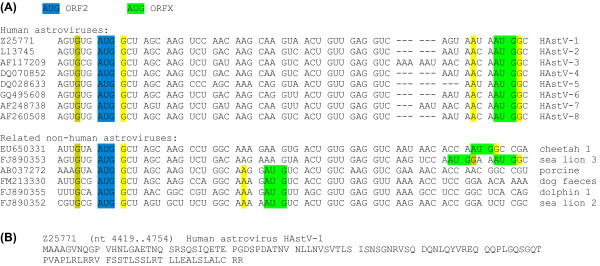
**Sequence data for mamastrovirus ORFX**. **(A) **Representative initiation codon contexts for mamastrovirus ORF2 and ORFX. Spaces separate ORF2-frame codons. Colour coding is as follows: blue - ORF2 initiation codon; green - potential ORFX initiation codon; yellow (olive) - flanking nucleotides matching the optimal (suboptimal) Kozak context. **(B) **Representative ORFX amino acid sequence.

ORFX appears amenable to translation via leaky scanning (Figure 2). Although the ORF2 AUG initiation codon generally has a 'G' at *-*3 and a 'G' at +4 (strong though not optimal Kozak context), it also appears to be positioned very close to the 5' end of the sgRNA (e.g. 12 nt in human astrovirus; [15]), and it has been shown that efficient leaky scanning can occur irrespective of Kozak context when an AUG codon is positioned within approximately 12 nt of the 5' end of an mRNA [16,17]. The ORFX AUG codon is typically 41 to 50 nt downstream and has an 'A' at *-*3 and a 'G' at +4 (strong Kozak context; conserved in 149 out of 152 human astrovirus sequences with coverage of both the ORF2 and ORFX AUG codons). In the porcine, dog and dolphin astrovirus sequences, the ORFX AUG codon is just 20 nt downstream of the ORF2 AUG codon but has an 'A' at *-*3 and a 'U' at +4 (medium context). In all cases, there are no intervening AUG codons in any frame. Interestingly, a product of the predicted size (12 kDa in human astrovirus) was observed when sequence corresponding to the sgRNA was translated *in vitro *[18].

The infectivity of a mutant astrovirus in which ORFX expression was inadvertently abolished was reduced by only 50% relative to wild-type virus [19] (a reduction which may be due to amino acid changes in the polyprotein frame besides the absence of ORFX product), thus demonstrating that the putative ORFX product is non-essential - at least for replication in cell culture. However, this does not imply that ORFX is not a CDS since the conditions or functions for which the presumed ORFX product is important may not have been directly tested (e.g. for comparison, the infectivity of a mutant alphavirus in which expression of the experimentally verified TF protein was abolished was also reduced by only ~50% [4]).

### Seadornavirus segment 7

**Figure 3:**
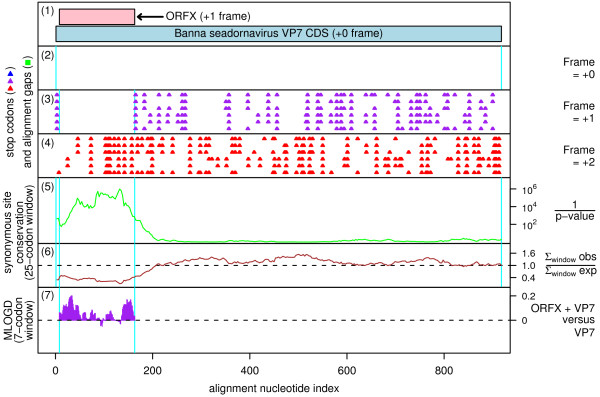
**Coding potential statistics for the seadornavirus VP7 CDS and the overlapping ORFX**. **(1) **Map of the VP7 CDS of Banna virus [GenBank: AF052018], showing the proposed new coding sequence, ORFX, overlapping the VP7 CDS in the +1 reading frame. **(2-7) **Coding potential statistics based on an alignment of six Banna virus sequences with complete coverage of the VP7 CDS (see Figure 4 for accession numbers). **(2-4) **Positions of stop codons in each of the three forward reading frames. Note the conserved absence of stop codons in the +1 frame within the ORFX region. **(5-6) **Conservation at synonymous sites within the VP7 CDS (see Figure 1 caption for details). Note the unusually high conservation within the ORFX region. **(7) **MLOGD statistics for ORFX (see [2] for details). The null model is that the sequence in the ORFX region is only coding in the +0 (VP7 CDS) frame, while the alternative model is that the ORFX region is coding in both the +0 and the +1 (ORFX) reading frames. Positive scores favour the alternative model. MLOGD coding potential scores are produced for each alignment column and averaged over a 21 nt sliding window for clarity. The predominantly positive scores indicate that ORFX is likely to be a coding sequence.

**Figure 4:**
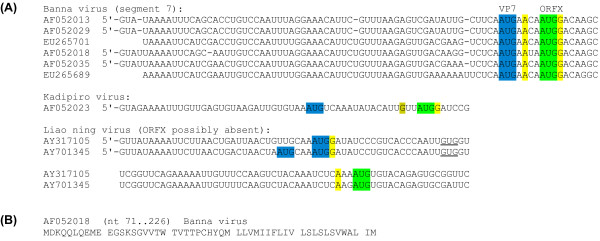
**Sequence data for seadornavirus ORFX**. **(A) **Initiation codon contexts for the seadornavirus segment 7 VP7 CDS and ORFX. Colour coding is as follows: blue - VP7 initiation codon; green - potential ORFX initiation codon; yellow (olive) - flanking nucleotides matching the optimal (suboptimal) Kozak context. **(B) **Representative ORFX amino acid sequence.

The seadornaviruses (family *Reoviridae*) are dsRNA viruses with 12 genome segments, all of which have so far been presumed to be monocistronic. These viruses are transmitted by mosquitoes and the type species, Banna virus (BAV), has been associated with fever, flu-like symptoms and encephalitis in infected humans [20,21]. ORFX overlaps the 5'-terminal region of the major CDS of segment 7 (which encodes VP7, a non-structural protein of uncertain function [22,23]) in the +1 reading frame. A 52-codon AUG-initiated +1 frame ORF is present in all available BAV sequences with complete coverage of the VP7 CDS (six sequences; two additional sequences have only partial coverage of ORFX; 16 Nov 2009). Application of MLOGD to the ORF reveals a strong coding signal (Figure 3). Similarly, there is greatly enhanced conservation at VP7-frame synonymous sites within the ORFX region (Figure 3). Although there is also enhanced conservation at the 5' end of other seadornavirus segments, the 3'-extent of the conservation is much greater in segment 7 than other segments. In all cases, the VP7 CDS utilizes the first AUG codon, which has a poor Kozak context ('U' at *-*3, 'A' at +4), while ORFX utilizes the second AUG codon, which is separated from the first AUG codon by just 4 nt and has a strong Kozak context ('A' at *-*3, 'G' at +4; Figure 4). Thus ORFX is amenable to translation via leaky scanning. Moreover, the close proximity of the ORFX AUG codon to the VP7 AUG codon may further enhance initiation at the latter, via the translation initiation coupling mechanism described in Ref. [24].

There are currently two other seadornavirus species with sequence coverage of the ORFX region - Kadipiro virus (KDV; 1 sequence), and Liao ning virus (LNV; 2 sequences) [20,25]. When MLOGD was applied to an alignment of the three species BAV, KDV and LNV, the results were ambiguous due to the high divergence between the different sequences. However, there is the potential, at least, for a functional ORFX in KDV, and possibly also LNV. In KDV, the VP7 CDS utilizes the first AUG codon, which has a weak Kozak context, while the second AUG (separated from the first AUG by 16 nt) is in the +1 reading frame and heads a 65-codon potential ORFX (Figure 4). In LNV, however, the VP7 CDS has two closely spaced AUG codons in one sequence, and a medium Kozak context in both sequences ('G' at +4), so is sub-optimal for leaky scanning. Moreover, although the next AUG codon is in the +1 reading frame, it is 64 nt downstream and only heads a 42-codon ORF. Thus, although there is a very strong case for a coding ORFX in BAV, whether or not this ORF is also present in KDV, and especially in LNV, can not be reliably assessed with the currently available sequence data.

### Cytorhabdovirus (Lettuce necrotic yellows and Lettuce yellow mottle viruses)

**Figure 5:**
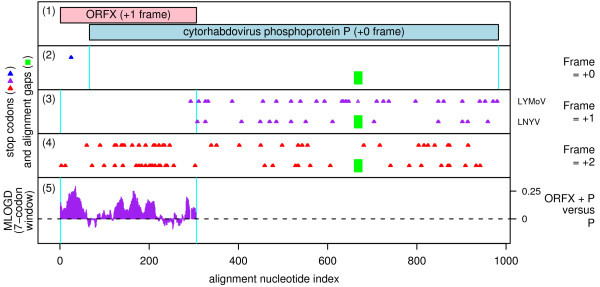
**Coding potential statistics for the cytorhabdovirus P CDS and the overlapping ORFX**. **(1) **Map of the P CDS of LNYV [GenBank: AJ867584], showing the proposed new coding sequence, ORFX, overlapping the P CDS in the +1 reading frame. **(2-5) **Coding potential statistics based on an alignment of LNYV and LYMoV (see Figure 6 for accession numbers). **(2-4) **Positions of stop codons in each of the three forward reading frames. Note the conserved absence of stop codons in the +1 frame within the ORFX region. **(5) **MLOGD statistics for ORFX (see Figure 3 for details). The predominantly positive scores indicate that ORFX is likely to be a coding sequence.

The cytorhabdoviruses comprise a genus within the family *Rhabdoviridae*, members of which have monopartite negative-sense ssRNA genomes. Species include Lettuce necrotic yellows virus (LNYV), Lettuce yellow mottle virus (LYMoV) and Northern cereal mosaic virus (NCMV). The genomes of LNYV and LYMoV contain at least six CDSs (N, P, putative movement protein, M, G, L) while the genome of the much more distantly related NCMV contains additional ORFs inserted upstream of M [26-28]. The 97-102 codon ORFX overlaps the 5' end of the P (phosphoprotein) CDS in the +1 reading frame and is present in LNYV and LYMoV but apparently not in NCMV (Figure 5).

**Figure 6:**
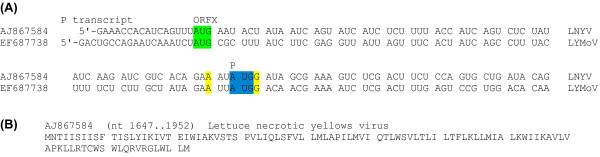
**Sequence data for cytorhabdovirus ORFX**. **(A) **Initiation codon contexts for the cytorhabdovirus P CDS and ORFX. Spaces separate ORFX-frame codons. Colour coding is as follows: blue - P initiation codon; green - potential ORFX initiation codon; yellow - flanking nucleotides matching the optimal Kozak context. **(B) **Representative ORFX amino acid sequence.

In fact these two sequences are the only two distinct sequences with coverage of ORFX currently available in GenBank (2 Nov 2009). The mean nucleotide identity between the two sequences within the ORFX region is only ~50%, which is below the ideal range for MLOGD (substitution saturation at high divergences makes it more difficult for MLOGD to distinguish between the single- and dual-coding models and causes a high-divergence 'turnover' in the MLOGD score [1]). Nonetheless, and notwithstanding the very limited sequence data, there is a good coding signal for ORFX (Figure 5). In fact the presence of this ORF has already been noted in both viruses [27,28] (and designated P' by Ref. [28]) though, so far as we are aware, this is the first evidence (apart from its conserved presence) that it is likely to be coding.

CDSs in the *Rhabdoviridae *are translated from a series of mRNA transcripts produced via a transcription termination-reinitiation mechanism, with conserved junction sequences containing the transcription stop and start signals located between consecutive CDSs so that mRNAs are generally monocistronic [26,28,29]. In the case of the P mRNA of LNYV and LYMoV, the P CDS utilizes the second AUG codon on the mRNA while the first AUG codon is in the correct frame for ORFX translation (Figure 6). However, the ORFX AUG codon has poor Kozak context ('U' at *-*3, 'A' or 'C' at +4; cf. [30]), which presumably allows a significant proportion of ribosomes to translate the P CDS via leaky scanning.

A similarly positioned overlapping CDS (generally referred to as 'C' and generally initiating downstream rather than upstream of the P initation site) occurs in certain paramyxovirus genera (e.g. *Morbillivirus*, *Respirovirus*) besides Vesicular stomatitis virus (*Vesiculovirus*, family *Rhabdoviridae*), though the C gene is likely to have arisen independently in the two families [31]. It has been suggested that the highly variable nature of the P protein facilitates the evolution of novel genes overlapping its N-terminal regions and, in the absence of discernable sequence homology, it cannot be assumed that the resulting proteins have similar functions [31,32].

### Coronaviruses

Coronaviruses (family *Coronaviridae*) belong to the order *Nidovirales*. At 26-32 kb, coronavirus genomes are among the largest of all RNA viruses. As with other members of the order, these viruses have a monopartite positive-sense ssRNA genome encoding a large replicase polyprotein that is expressed from the genomic RNA (ORF1a and, via ribosomal frameshifting, an ORF1a-ORF1b fusion product), and a number of other proteins which are translated from a nested set of 3'-coterminal sgRNAs [33,34]. The coronaviruses are currently classified into three main groups (recently elevated to genera) which are further divided into subgroups, though there are also a large number of species that await formal classification. Although a core set of sgRNA-encoded structural proteins (S, E, M, N) are conserved throughout all groups, a variable number of auxilliary proteins are also encoded by sgRNAs - including a number of known overlapping CDSs (e.g. the I CDS that overlaps the N CDS of some Group 2 coronaviruses [35]). We have identified two new candidates - one in Group 3 coronaviruses of the proposed subgroup 3c [36], and one in certain Group 1b coronaviruses. (Note that, while the evidence for the coding status of the preceding three candidates is very strong, the evidence for these two candidates is less certain but should become clearer as more sequence data become available.)

### Coronavirus Group 3c

**Figure 7:**
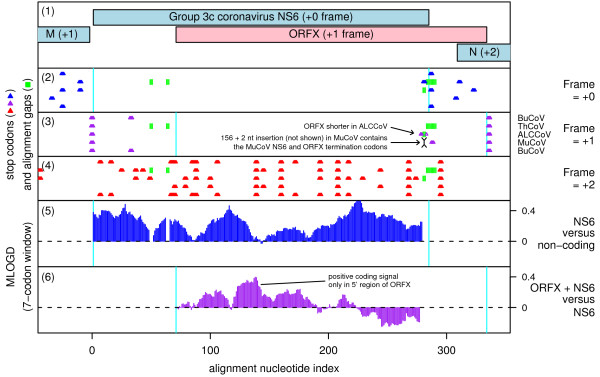
**Coding potential statistics for the Group 3c coronavirus NS6 CDS and the overlapping ORFX**. **(1) **Map of the NS6 CDS of BuCoV [GenBank: FJ376620], showing the proposed new coding sequence, ORFX, overlapping the NS6 CDS in the +1 reading frame. **(2-5) **Coding potential statistics based on an alignment of five Group 3c coronavirus sequences (see Figure 8 for accession numbers). **(2-4) **Positions of stop codons in each of the three forward reading frames. Note the conserved absence of stop codons in the +1 frame within the ORFX region. **(5) **MLOGD statistics for NS6 relative to a non-coding null model. **(6) **MLOGD statistics for ORFX (see Figure 3 for details). The predominantly positive scores indicate that ORFX is likely to be a coding sequence, but is subject to significantly weaker purifying selection than NS6. The negative scores at the 3' end of ORFX indicate that the C-terminal region of the putative product is not subject to strong functional constraints.

In the subgroup 3c coronaviruses, ORFX overlaps the NS6 CDS (between M and N) in the +1 reading frame. ORFX appears to be present in all available (8 Nov 2009) subgroup 3c sequences with coverage of the NS6 region: Thrush coronavirus (ThCoV), Bulbul coronavirus (BuCoV; 2 sequences), Munia coronavirus (MuCoV) and Asian leopard cat coronavirus (Figure 7) [36,37]. ORFX has length 69-89 codons and covers 73-81% of NS6 (91-108 codons).

**Figure 8:**
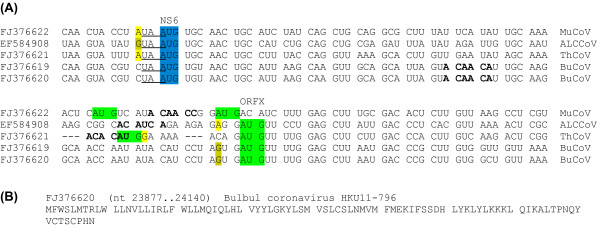
**Sequence data for Group 3c coronavirus ORFX**. **(A) **Initiation codon contexts for the Group 3c coronavirus NS6 CDS and ORFX. Spaces separate NS6-frame codons. Colour coding is as follows: blue - NS6 initiation codon; green - potential ORFX initiation codon; yellow (olive) - flanking nucleotides matching the optimal (suboptimal) Kozak context. Potential, albeit imperfect, TRSs are indicated in bold. The termination codon of the upstream M CDS is underlined. **(B) **Representative ORFX amino acid sequence.

ORFX appears amenable to translation via leaky scanning from the same sgRNA as NS6 since the NS6 AUG initiation codon has a 'G', 'A' or 'C' at *-*3 (depending on species) and a 'U' at +4 for a medium or weak Kozak context (Figure 8) and there are no intervening AUG codons in any frame between the NS6 AUG codon and the ORFX AUG codon. However, ORFX translation may be a little more complex since Ref. [36] were unable to identify a transcription regulatory sequence (TRS; ACACCA in these viruses) for production of an NS6 sgRNA, which may mean that NS6 is translated via some non-canonical mechanism (such as reinitiation on the M sgRNA; cf. [38]; in fact the UAA termination codon of the M CDS overlaps the AUG initiation codon of the NS6 CDS as UAAUG; Figure 8). Potential, albeit imperfect, TRSs between the NS6 and ORFX initiation codons (Figure 8) may mean that a separate sgRNA is produced for ORFX. Although Ref. [36] were unsure about the coding status of NS6 itself (due to the apparent absence of a TRS, and a relatively high *K*_a_/*K*_s _ratio), our MLOGD analysis suggests that NS6 is coding and is subject to significantly stronger functional constraints than ORFX (Figure 7, panel 5).

### Bat coronaviruses 1A, 1B, HKU8

**Figure 9:**
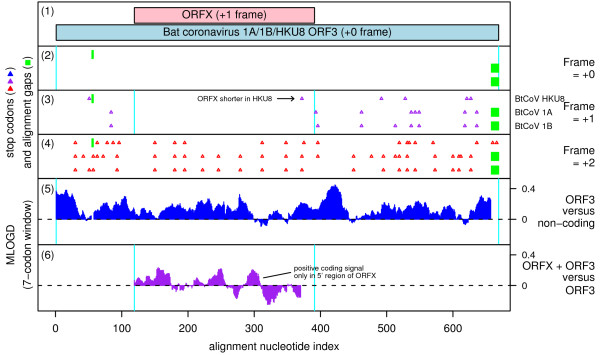
**Coding potential statistics for bat coronavirus 1A/1B/HKU8 ORF3 and the overlapping ORFX**. **(1) **Map of the ORF3 region of BtCoV 1A [GenBank: EU420138], showing the proposed new coding sequence, ORFX, overlapping ORF3 in the +1 reading frame. **(2-5) **Coding potential statistics based on an alignment of BtCoV 1A, 1B and HKU8 (see Figure 10 for accession numbers). **(2-4) **Positions of stop codons in each of the three forward reading frames. Note the conserved absence of stop codons in the +1 frame within the ORFX region. **(5) **MLOGD statistics for ORF3 relative to a non-coding null model. **(6) **MLOGD statistics for ORFX (see Figure 3 for details). The predominantly positive scores indicate that ORFX is likely to be a coding sequence, but is subject to significantly weaker purifying selection than ORF3. The negative scores at the 3' end of ORFX indicate that the C-terminal region of the putative product is not subject to strong functional constraints.

**Figure 10:**
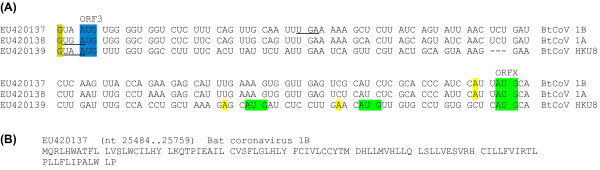
**Sequence data for bat coronavirus 1A/1B/HKU8 ORFX**. **(A) **Initiation codon contexts for bat coronavirus 1A/1B/HKU8 ORF3 and ORFX. Spaces separate ORF3-frame codons. Colour coding is as follows: blue - ORF3 initiation codon; green - potential ORFX initiation codon; yellow (olive) - flanking nucleotides matching the optimal (suboptimal) Kozak context. The termination codon of the upstream S CDS is underlined. **(B) **Representative ORFX amino acid sequence.

Here, ORFX overlaps ORF3 (between S and E) in the +1 reading frame (Figure 9). ORFX appears to be present in bat coronavirus 1B (BtCoV 1B), bat coronavirus 1A (BtCoV 1A), and bat coronavirus HKU8 (BtCoV HKU8) [39] (just three sequences available), but does not appear to have a wider phylogenetic distribution among sequences currently available in GenBank (15 Nov 2009). ORFX appears amenable to translation via leaky scanning from the same sgRNA as ORF3 since the ORF3 AUG initiation codon has a 'G' at *-*3 and a 'U' at +4 for a medium to weak Kozak context, and there are no intervening AUG codons in any frame between the ORF3 AUG codon and the ORFX AUG codon (Figure 10). ORFX has length 91-95 codons. As with Group 3c coronavirus NS6, our MLOGD analysis also confirmed the probable coding status of ORF3, and showed that ORF3 is subject to significantly stronger functional constraints than ORFX (Figure 9, panel 5).

## Conclusions

Overlapping genes are difficult to identify and are often overlooked. However, it is important to be aware of such genes as early as possible in order to avoid confusion (otherwise functions of the overlapping gene may be wrongly ascribed to the gene they overlap), and also so that the functions of the overlapping gene may be investigated in their own right. Computational analysis of sequence data is a time- and cost-efficient way to find such genes and help direct experimental follow-up.

The list of new candidate overlapping genes presented here is by no means complete as we have omitted several candidates that we are currently following up experimentally, and a number of candidates that are less certain - for example candidates where a conserved potential translation mechanism has not been identified, or candidates where a significant fraction of isolates contain premature termination codons, or candidates with too little phylogenetic support within the currently available sequence databases. To the best of our knowledge (except as noted for cytorhabdovirus) these candiates have not previously been described or annotated elsewhere, and we apologize if we have accidently omitted any previous references to any of these candidates.

## Methods

Virus sequences were downloaded from GenBank and alignments were generated using standard bioinformatics software (blast [40], clustal [41] and EMBOSS [42]). Candidate overlapping CDSs were identified using either MLOGD or analysis of conservation at synonymous sites of the annotated CDSs as described previously [1,2,5]. The following astrovirus sequences with complete coverage of ORF2 were used for the alignment and statistics illustrated in Figure 1: [GenBank: AB000283, AB000284, AB000285, AB000286, AB000287, AB000288, AB000289, AB000290, AB000291, AB000292, AB000293, AB000294, AB000295, AB000296, AB000297, AB000298, AB000299, AB000300, AB000301, AB009984, AB009985, AB013618, AB025801, AB025802, AB025803, AB025804, AB025805, AB025806, AB025807, AB025808, AB025809, AB025810, AB025811, AB025812, AB031030, AB031031, AB037272, AB037273, AB037274, AB290149, AB308374, AB496913, AF056197, AF117209, AF141381, AF248738, AF260508, AY720891, AY720892, DQ028633, DQ070852, DQ344027, DQ630763, EF138823, EF138824, EF138825, EF138826, EF138827, EF138828, EF138829, EF138830, EF138831, EF583300, FJ755402, FJ755403, FJ755404, FJ755405, FJ890352, FJ890355, FM213330, FM213331, FM213332, GQ405855, GQ405856, GQ405857, GQ495608, GQ901902, L06802, L13745, L23513, S68561, U15136, Y08632, Y15938, Z25771, Z33883, Z46658, Z66541]. The seadornavirus, cytorhabdovirus and coronavirus sequences used are listed in Figures 4, 6, 8 and 10. Additional sequences with partial coverage of the annotated CDS which each overlapping gene candidate overlaps were available for the seadornaviruses ([GenBank: EU265679] and [GenBank: EU265722]) and the mamastroviruses (~700 sequences; not listed).

## Competing interests

The authors declare that they have no competing interests.

## Authors' contributions

AEF carried out the bioinformatic analysis and wrote the manuscript. Both authors edited and approved the final manuscript.
